# Integrin-Linked Kinase Expression in Human Valve Endothelial Cells Plays a Protective Role in Calcific Aortic Valve Disease

**DOI:** 10.3390/antiox11091736

**Published:** 2022-08-31

**Authors:** Sandra Sánchez-Esteban, Mercedes Castro-Pinto, Alberto Cook-Calvete, Paula Reventún, María Delgado-Marín, Lucía Benito-Manzanaro, Ignacio Hernandez, José López-Menendez, José Luis Zamorano, Carlos Zaragoza, Marta Saura

**Affiliations:** 1 Universidad de Alcalá, Dpto Biología de Sistemas/Fisiología, IRYCIS, 28871 Alcalá de Henares, Spain; 2 Centro de Investigación Biomédica en Red de Enfermedades Cardiovasculares (CIBERCV), Instituto de Salud Carlos III, 28034 Madrid, Spain; 3 Hospital Ramón y Cajal, Servicio Cirugía Cardiaca Adultos, 28034 Madrid, Spain; 4 Hospital Ramón y Cajal, Unidad de Investigación Cardiovascular, Instituto Ramón y Cajal de Investigación Sanitaria (IRYCIS), Universidad Francisco de Vitoria, Pozuelo de Alarcón, 28223 Madrid, Spain; 5 Hospital Universitario Ramón y Cajal, Servicio de Cardiología, Instituto Ramón y Cajal de Investigación Sanitaria (IRYCIS), 28034 Madrid, Spain; 6 Division of Cardiology, Department of Medicine, School of Medicine, Johns Hopkins University, Baltimore, MD 21205, USA

**Keywords:** ILK, nitric oxide, EndMT, calcific valve disease, endothelial dysfunction, aging

## Abstract

Calcific aortic valve disease (CAVD) is highly prevalent during aging. CAVD initiates with endothelial dysfunction, leading to lipid accumulation, inflammation, and osteogenic transformation. Integrin-linked kinase (ILK) participates in the progression of cardiovascular diseases, such as endothelial dysfunction and atherosclerosis. However, ILK role in CAVD is unknown. First, we determined that ILK expression is downregulated in aortic valves from patients with CAVD compared to non-CAVD, especially at the valve endothelium, and negatively correlated with calcification markers. Silencing ILK expression in human valve endothelial cells (siILK-hVECs) induced endothelial-to-mesenchymal transition (EndMT) and promoted a switch to an osteoblastic phenotype; SiILK-hVECs expressed increased RUNX2 and developed calcified nodules. siILK-hVECs exhibited decreased NO production and increased nitrosative stress, suggesting valvular endothelial dysfunction. NO treatment of siILK-hVECs prevented VEC transdifferentiation, while treatment with an eNOS inhibitor mimicked ILK-silencing induction of EndMT. Accordingly, NO treatment inhibited VEC calcification. Mechanistically, siILK-hVECs showed increased Smad2 phosphorylation, suggesting a TGF-β-dependent mechanism, and NO treatment decreased Smad2 activation and RUNX2. Experiments performed in eNOS KO mice confirmed the involvement of the ILK-eNOS signaling pathway in valve calcification, since aortic valves from these animals showed decreased ILK expression, increased RUNX2, and calcification. Our study demonstrated that ILK endothelial expression participates in human CAVD development by preventing endothelial osteogenic transformation.

## 1. Introduction

Calcific aortic valve disease (CAVD) has been described as the most common valve disease and the third most common cause of cardiovascular disease worldwide [1]. The global prevalence is 12.6 million cases per year, affecting about 2% of people over 65 years of age—a rate that will increase in the coming decades due to population aging [2]. CAVD is a highly active ossification process, in which the accumulation of calcium deposits in the aortic valve leaflets impairs the proper outflow of blood from the left ventricle, leading to heart failure [3]. The main clinical challenge of CAVD is early detection to manage the disease from its early stages. Numerous promising pharmacological treatments have emerged in recent years, but aortic valve replacement (surgical or interventional) remains the only effective treatment, leaving the way open for new therapeutic targets [4].

The valve cusps are covered by valve endothelial cells (VECs) that serve as a physical barrier between valve interstitial cells (VICs) and the blood flow and are crucial in maintaining valvular integrity and homeostasis. Several stimuli, mainly aberrant blood flow patterns and shear stress, activate the capacity of the VECs and VICs to undergo transformation from physiological cell types to a pathological phenotype through a variety of cellular and molecular responses that culminate in AoV calcification [1]. The onset of the disease is characterized by a loss of valve endothelial cell integrity, leading to endothelial dysfunction and, as a result, lipid accumulation, inflammation, and even cell transformation through the endothelial-to-mesenchymal transition process (EndMT) [3,5]. During EndMT, endothelial cells lose endothelial-specific markers (CD31 and VE-Cadherin) and acquire myofibroblast markers (α-smooth muscle actin and transgelin). Many studies suggest that VECs undergoing EndMT may differentiate into osteoblastic interstitial cells in a TGF-β-dependent manner [6,7]; however, the role of VECs in the osteogenic process has not been fully explored.

Integrin-linked kinase (ILK) is a protein involved in preserving endothelial function. ILK, a serine/threonine kinase that binds to the cytoplasmic domain of β-Integrins, is an essential mechanosensitive protein widely expressed in the cardiovascular system that regulates several biological processes, such as angiogenesis, cell proliferation, survival, migration, and epithelial–mesenchymal transition (EMT) [8,9]. We previously demonstrated that ILK regulates vasomotor function by preventing endothelial nitric oxide synthase (eNOS) uncoupling and promoting physiological nitric oxide (NO) production [10], and ILK expression can be decreased at the endothelium in proinflammatory conditions and during atherosclerosis [11]. Thus, we hypothesize that endothelial ILK plays a protective role in calcific aortic valve disease, preventing valve endothelial dysfunction and osteogenic cell differentiation in a nitric oxide-dependent manner.

## 2. Materials and Methods

A detailed listing of the reagents and antibodies used in the study is provided in Table S1.

### 2.1. Human Calcified Aortic Valves

The human study was conducted in collaboration with the Department of Cardiac Surgery in Ramón y Cajal Hospital in Madrid, Spain. Aortic valve leaflets were harvested from patients undergoing valve replacement for aortic valve stenosis (CAVD) and aortic insufficiency without calcific lesions (non-CAVD). Human samples were collected as surgical residues with the approval of the Ramon y Cajal Hospital Ethics Committee (CEIC 17 December 2018) and after informed consent of the patients. The demographic and clinical characteristics were collected at the time of the intervention in the database of the Cardiac Surgery service of the Ramón y Cajal University Hospital. Both types of aortic valve tissues were processed identically: of the three leaflets collected, one was processed in 10% formalin for histological study, the second was stored at −80 °C for protein analysis, and the third was cultured for valve endothelial cell (hVEC) isolation.

### 2.2. Animals

WT C57BL/6 mice and (C57BL/6,129) eNOS null mice (eNOS KO mice) were purchased from The Jackson Laboratory and housed in our own animal facilities in isolated rooms. All animal research was carried out following the recommendations of our institutional animal welfare officer at Universidad de Alcalá and the Regional Administrative Council of Comunidad de Madrid under the code PROEX 231.2/20.

### 2.3. Histological Detection of Calcium Deposits

As previously described, human valve tissues were processed in 10% formalin [12]. Sections (5 μm) were deparaffinized in xylene, rehydrated, and stained with a Von Kossa staining kit (abcam) or Alizarin Red Solution 40 nM (Sigma-Aldrich, San Luis, MO, USA) to explore calcium deposits. Images were quantified from at least 4 high-power fields per section.

Calcium deposits in hVECs were detected as previously described [6]. At least four images were taken for every experimental condition using NIS element D3.2 Nikon software (Nikon, Tokyo, Japan) and quantified with ImageJ software.

### 2.4. Immunohistochemistry

After xylene deparaffinization, tissue sections were boiled in citrate buffer for antigen retrieval. Then, following the manufacturer’s instructions for the Mouse and Rabbit Specific HRP/DAB (ABC.) Detection IHC kit (Abcam, Cambrige, UK), we incubated primary antibodies overnight at 4 °C. Once the protocol was complete, sections were washed and counterstained with Carazzi hematoxylin, dehydrated, and mounted with DPX (Casa Alvarez, Madrid, Spain) [13]. Several images from 2 different valve sections (n = 6 valves/group) were taken using a Nikon brightfield microscope under 40× magnification and quantified with ImageJ 2.0.0 software (NIH, Bethesda, MD, USA).

### 2.5. Confocal Microscopy

Tissue sections (5 μm) were deparaffinized and rehydrated for subsequent permeabilization in citrate buffer. After 30 min of BSA protein blocking, tissue was incubated with the primary antibodies overnight at 4 °C. After washing with PBS, the slides were incubated with FITC, Alexa-488-, or Alexa-647-conjugated secondary antibodies, for 1 h at room temperature. Sections were washed twice with PBS and then mounted with Hoechst. Images were taken for data quantification using a Leyca TCS SP5 confocal microscope (UAH-NANBIOSIS-CIBER-BNN, Madrid, Spain). The fluorescence-conjugated secondary antibodies used were Alexa-488 (excitation wavelength, 543 nm; emission wavelength, 586–590 nm) and Alexa-647 (excitation wavelength, 650 nm; emission wavelength, 665 nm) both from Abcam (Cambridge, UK). Nuclei were stained with Hoechst (excitation wavelength, 405 nm; emission wavelength, 424 nm) from Abcam (Cambridge, UK). Fluorescent images were captured at × 60 magnification.

### 2.6. Human Valve Endothelial Cell (hVEC) Isolation

The surgical team collected human aortic valves (CAVD and non-CAVD) in DMEM/F12 culture medium supplemented with 10% Fetal Bovine Serum (FBS) from Gibco (Waltham, MA, USA) and 0.05 mg/mL Penicillin/Streptavidin (Lonza, Basel, Switzerland), and carried them into a laminar flow hood. After several washes with PBS, the leaflets were cut into 2 mm pieces and seeded in a Matrigel (Corning)-coated culture dish. Valve explants were grown in EBM-2 medium supplemented with an EGM-2 kit (CC4176, Lonza, Basel, Switzerland) for two weeks. Then, explants were removed, and the endothelial cells were released from the Matrigel using 2 U/mL Dispase II solution (Roche) for 1 h at 37 °C. The cell suspension was centrifuged at 900 rpm for 5 min, and the cell pellet was seeded in a gelatin-coated plate. VECs were characterized by flow cytometry and confocal microscopy by double staining of CD31 and α-SMA. It was determined that 97,7% of the primary culture was positive for CD31 but negative for α-SMA, confirming the endothelial phenotype.

Isolated hVECs were cultured in EBM-2 medium supplemented with an EGM-2 kit (Lonza) in a humidified CO2 incubator with 5% CO_2_ at 37 °C. Cells from passages 2 to 5 were used for the experimental procedure [14].

### 2.7. Immunoblot

Protein lysates were processed as described in [10]. In brief, tissue was frozen and harvested in a protein lysis buffer (Novagen cytobuster protein extraction reagent (EMD chemicals, San Diego, CA, USA)) supplemented with Complete Mini and Phospho-stop reagents (Roche). Protein (15 μg) was separated via 10% SDS-polyacrylamide gel electrophoresis and transferred to a PVDF membrane (Biorad, Hercules, CA, USA). For protein detection, membranes were blocked for 1 h in 5% BSA in TTBS buffer and incubated with specific antibodies overnight at 4 °C. After several washes, the membranes were incubated with a secondary antibody. The immunoreactive bands were visualized with the Pierce™ ECL Western Blotting Substrate, according to the manufacturer’s procedures (Pierce, Waltham, MA, USA).

### 2.8. Cell Transfection

hVECs were transfected with 25 nM non-targeting siRNA (si-Scramble) or ILK-targeting siRNA (si-ILK) (Santa Cruz Biotech, Santa Cruz, CA, USA) using Lipofectamine 2000 transfection reagent and Opti-MEM (Gibco, Waltham, MA, USA) for 6 hours. Following transfection, cells were cultured in EBM-2 medium for 5 or 7 days, according to experimental conditions. Specific cell treatments were performed 24 h after transfection, following the protocol described in the corresponding section. HuVECs were transfected with an empty vector (pcDNA) or plasmids encoding wild-type ILK (ILK-WT) [10], using Lipofectamine 2000.

### 2.9. Cell Treatments

In order to mimic constant NO release in transfected hVECs, cells were treated with DETA NONOate (10^−7^ M) every 48 h in culture medium. eNOS activity was inhibited using L-NAME 1 mM.

The inhibition of the BMP2 pathway was achieved by supplementing the culture medium with the BMP antagonist Noggin (500 ng/mL) or with the selective inhibitor LDN-193189 (500 nM). The medium was changed every 2–3 days.

### 2.10. Pro-Osteogenic Culture Medium

To mimic an osteogenic environment in vitro, cells were cultured for up to 15 days with pro-osteogenic medium (DMEM with 10% FBS, 0.05 mg/mL penicillin/streptomycin, and 2.5 μg/mL amphotericin with 10 nmol/L beta-glycerolphosphate, 50 μmol/L ascorbic acid, and 10 μmol/L dexamethasone) or control medium (DMEM with 10% FBS, 0.05 mg/mL penicillin/streptomycin, and 2.5 μg/mL amphotericin). Media were replaced every 2–3 days. For some experiments, cells were transfected with siRNA, as indicated, on day eight and treated every two days with DETA NO NOate (10^−7^ M). In some experiments, hVECs were cultured for 21 days in pro-osteogenic medium.

### 2.11. Nitric Oxide and Superoxide Anion Production

DAF-2 reacts with NO to yield the highly fluorescent triazolofluorescein, while DHE reacts with superoxide to form 2-hydroxyethidium. For testing of eNOS Nitric oxide production, hVECs were seeded on a glass coverslip and siRNA was transfected as previously described. NO production was measured in cells loaded with DAF-DA diacetate (5 mM), and propidium iodide (1 µg/mL) was used to determine cell viability. After exposure to different experimental conditions—saline buffer or 10 nM Acetylcholine (ACh) for 15 min, or pretreatment with 1 mM L-NAME for 18 h and then ACh for 15 min—the cells were trypsin-dispersed and labeled with the fluorochromes at 37 °C, followed by cytofluorometric analysis. Data acquisition was performed on a MACSQuant 10 flow cytometer (Miltenyi Biotec), and the data were analyzed with MACSQuant software. Data were represented as relative fluorescent units (RFUs) to siSc or siILK control conditions. Some coverslips were treated in the same way and then washed with PBS and fixed in 2% paraformaldehyde for 20 min. Nuclei were stained with Hoechst, and glass coverslips were mounted in Fluorsave Reagent (Merck Millipore, Burlintong, MA, USA).

For the testing of superoxide anion production, transfected hVECs were incubated with saline buffer or 10 nM Acetylcholine (ACh) for 15 min in combination with 10 µM Apocynin for 1 h to inhibit endogenous NADPH oxidase formation of superoxide or ACh in combination with 1 mM L-NAME for 18 h. Cells were then incubated with 1 µM DHE for 15 min at 37 °C, washed, fixed, and mounted as before. Superoxide and Nitric Oxide were determined by confocal microscopy at 575–700 nm and 491–513 nm, respectively.

### 2.12. Statistical Analysis

All results were expressed as means ± standard deviations (SDs). GraphPad Prism 7.0 software (GraphPad Software Inc., San Diego, CA, USA) was used for statistical analysis. Every experimental condition was duplicated within each experiment, and each experiment was repeated at least three times. For animal studies, n values refer to the number of individual animals used. Statistical significance between groups was examined using the Student’s *t*-test or one-way ANOVA, according to the experimental requirements. For the human valve study, the Pearson test was used to correlate the continuous numerical variables with a normal distribution and the Spearman test was used for variables with a non-normal distribution. The Shapiro–Wilk test was used to confirm the normal distribution of the variables. Stepwise multiple logistic regression was performed using STATA 14 statistical software (Stata Corp LP, College Station, TX, USA). Unless otherwise stated, differences between groups were considered significant at *p*-values < 0,01 (* *p* < 0.01; ** *p* < 0.001; *** *p* < 0.0001).

## 3. Results

### 3.1. ILK Decrease in Aortic Endothelial Cells of Calcific Aortic stenosis Patients

We studied 103 CAVD patients (mean age of 67.3 years (SD, 12.6 years), 69.9% men; preserved left ventricular ejection fraction (LVEF) (51.5%), moderately depressed LVEF (21.3%), only five patients with severely depressed LVEF (4.8%)) from the Ramón y Cajal Hospital (Madrid, Spain) who required valve replacement. Seventy-three patients (70.87%) presented predominantly moderate–severe AS (mean gradient, 44.4 ± 16.1 mm Hg) and calcification (CAVD). Thirty patients (29.13%) with little or no calcification served as non-CAVD controls; 67.86% were men, with a mean age of 66.37 years (SD, 10.68). The most relevant clinical characteristics and preoperative comorbidities are presented in Supplementary Table S2. Of the patients with valve calcification (CAVD), the most prevalent comorbidities were hypertension (65.3%), diabetes mellitus (30.67%), and dyslipidemia (58.67%), and 49.33% were classified in NYHA functional classes III-IV. Of the patients without valve calcification (non-CAVD), the most prevalent comorbidities were hypertension (75%) and concomitant aortic aneurysm disease (35.71%). It was found that 50% were in NYHA functional classes III-IV.

**Figure 1:**
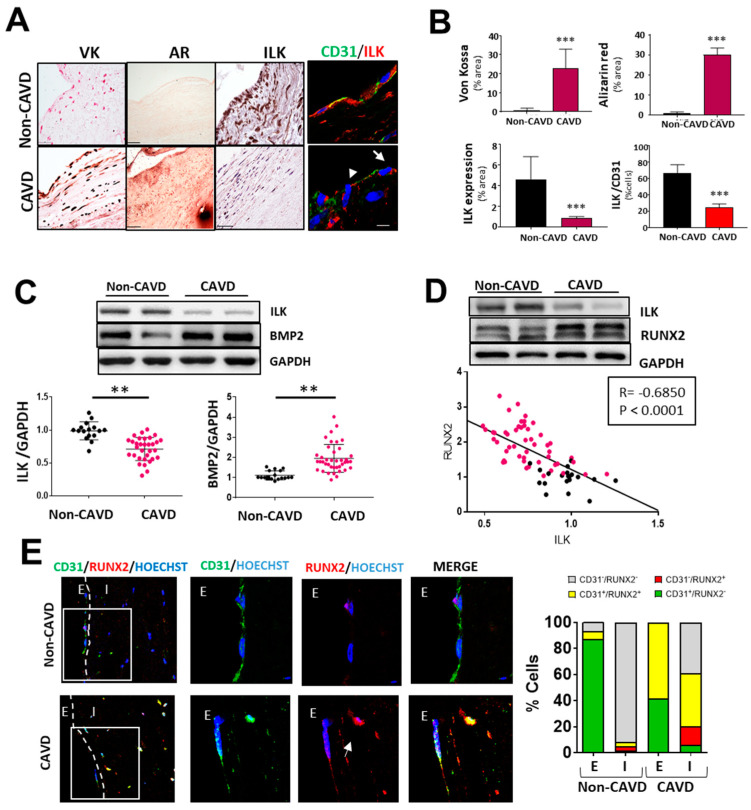
Endothelial ILK is downregulated in calcific aortic valve disease. (**A**) Von Kossa (VK) and Alizarin Red (AR) staining of calcium deposits (left panel); immunohistochemistry of ILK (central panel); and immunofluorescence of ILK in red and the endothelial marker CD31 in green (right panel) in non-CAVD or CAVD human aortic valve leaflets. Nuclei were counterstained with Hoechst. Arrows show valve endothelial cells. Scale bar = 25 µm. (**B**) Quantification of calcification and ILK expression shown in (**A**). n = 10. *** *p* < 0.001 vs. non-CAVD. (**C**,**D**) Western blot analysis of non-CAVD vs. CAVD valve tissue proteins. (**C**) ILK and BMP2 expression and quantification (n = 16–31, ** *p* < 0.01). (**D**) ILK and RUNX2 expression and Pearson correlation (R = −0.685, *** *p* < 0.0001, n = 72). (**E**) Confocal fluorescent microscopy images of CD31 (green) and RUNX2 (red) in non-CAVD and CAVD aortic leaflets. The dotted line delimits the endothelium (**E**) from the interstitium (I). Cell nuclei were counterstained with Hoechst. On the right, magnifications of areas enclosed in white squares. Scale bar = 25 µm. Arrowheads indicate cells within the valves expressing both markers. Percentages of cells expressing CD31, RUNX2, or both markers are shown on the right. n = 4.

First, we analyzed the protein expression of ILK in human valve samples collected as surgical residues from patients with calcific aortic valve stenosis and age-matched non-CAVD controls. CAVD specimens with evident calcific lesion formation as detected by Von kossa and Alizarin red staining exhibited decreased ILK protein expression. In contrast, ILK was abundantly expressed in non-CAVD controls. Since CAVD pathology starts at the endothelium level, where significant hemodynamic stress occurs [15], we focused on valve endothelia to study ILK expression. Co-immunostaining of ILK and the endothelial marker CD31 detected a blunted decrease in ILK at the endothelial level (Figure 1A,B). A more in-depth study confirmed a significant negative correlation between ILK expression and valve calcification (Figure 1C). In addition, BMP2, another protein widely implicated in calcification, showed a marked increase in CAVD vs. non-CAVD valves. To control for confounding factors, we used stepwise multiple logistic regression to create a multivariate model that included age, calcification degree, ILK levels, and comorbidities related to atherosclerosis and valvular calcification, such as diabetes, hypertension, and dyslipidemia. Significant values were those the probability of which was less than 5% (*p* < 0.05). ILK presented a significant association with valve calcification (adjusted OR, 0.0096; SD, 0.157; *p* < 0.005), with dyslipidemia being equally relevant (OR, 4.63; SD, 2.71; *p* < 0.009). In this case, ILK proved to be a protective factor. Indeed, the expression levels of RUNX2, a marker of calcification, showed a significant negative linear correlation with ILK (Pearson’s linear correlation coefficient, −0.68; *p* < 0.001) (Figure 1D). Together these results showed a negative correlation between ILK expression and valvular calcification.

Moreover, in non-CAVD valves, RUNX2 expression was absent in both endothelial CD31^+^-VECs covering the leaflets and VICs. However, in aortic valve leaflets with advanced CAVD, RUNX2 expression could be detected in CD31^+^-endothelial cells (58.33 ± 2.12%), and 39.08 ± 1.23% of valve interstitial cells expressed both CD31 and RUNX2 proteins, suggesting endothelial cell transdifferentiation (Figure 1E).

### 3.2. ILK Prevents EndMT and the Osteogenic Phenotype Switch in hVECs

**Figure 2:**
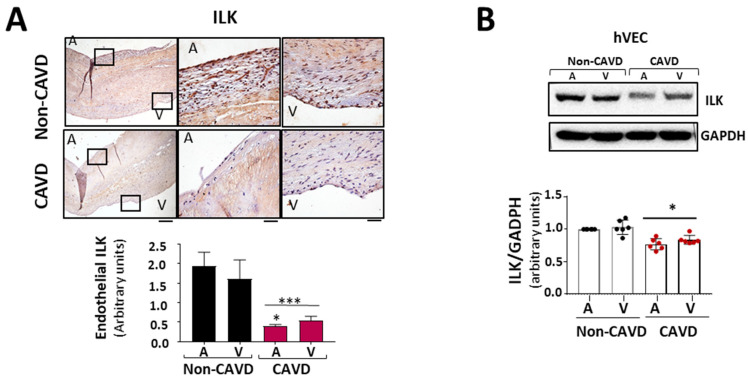

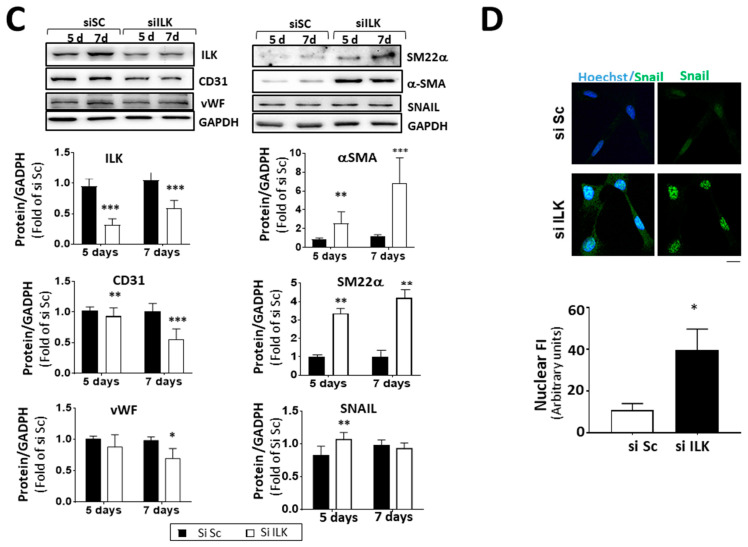
ILK prevents endothelial-to-mesenchymal transition (EndMT) in human valve endothelial cells (hVECs). (**A**) Immunohistochemistry of ILK in the aortic and ventricular sides of the endothelial layers of non-CAVD or CAVD human aortic valve leaflets. Central and right panels are magnifications of the areas enclosed in squares in the left panels. A = aortic side, V = ventricular side. Scale bar = 100 µm (left panel); 10 μm (central and right panels). Quantification of ILK expression is shown below. n = 6. *** *p* < 0.001 vs. non-CAVD; * *p* < 0.01 vs. V side CAVD. (**B**) Western blot and quantification of ILK protein expression in isolated non-CAVD or CAVD human valve endothelial cells (hVECs). Each point represents ILK expression in VECs isolated from different patients. n = 6. * *p* < 0.01 vs. non-CAVD. (**C**) Western blot analysis and quantification of ILK and the EndMT markers CD31, von Willebrand Factor (vWF), alpha-smooth muscle actin (α-SMA), transgelin (SM22α), and Snail in hVECs transfected with siRNA Scramble (si Sc) or siRNA ILK (si ILK) for 5 or 7 days. n = 11, CD31, αSMA, and ILK; n = 6, Snail, SM22α, and vWF. * *p* < 0.01 vs. Si Sc; ** *p* < 0.001 vs. Si Sc; *** *p* < 0.001 vs. Si Sc. (**D**) Confocal fluorescent microscopy images (left) and quantification of nuclear fluorescence intensity (Nuclear FI) (right) of Snail (green) in transfected hVECs for 5 days. Cell nuclei were counterstained with Hoechst. Scale bar = 10 µm. n = 6. * *p* < 0.01 vs. Si Sc.

Since the aortic side of the Ao valve is the preferential site of calcification [15], we analyzed ILK expression in the endothelial layer of both sides of the valve, aortic and ventricular. Immunohistochemically, ILK staining showed decreased expression of ILK at both sides of the endothelium in CAVD vs. non-CAVD samples, but ILK expression decreased more on the aortic side (Figure 2A). To better study these phenomena, we isolated endothelial valve cells (VECs) from the aortic and ventricular sides to study them separately. Both VEC types expressed lower ILK levels compared to non-CAVD controls. However, endothelial cells isolated from the aortic side of CAVD patients exhibited lower ILK expression than those isolated from the ventricular side (Figure 2B), suggesting a possible role for ILK in the early stage of this process. Thus, hVECs isolated from the aortic side of non-calcific samples were used to study the mechanism associated with ILK loss during CAVD. 

Endothelial-to-mesenchymal transition (EndMT) plays a role in the pathogenesis of CAVD [5,16]. ILK silencing for five days increased the expression and nuclear accumulation of transcription factor Snail (SNAI1), crucial for dissolution of E-cadherin junctions during EndMT, suggesting a role for ILK in the EndMT process (Figure 2C,D). Moreover, ILK silencing in hVECs (siILK-hVECs) resulted in the expression of mesenchymal cell markers α-SMA and SM22α, five and seven days after ILK depletion. At the same time, there was a progressive loss of endothelial cell markers CD31 and vWF, which was more evident seven days after ILK silencing (Figure 2D).

Several reports suggested that activated VECs might cooperate with activated VICs to promote CAVD progression via the EndMT process; endothelial-derived VICs (eVICs) might further differentiate into osteogenic VICs and contribute to calcification in CAVD, characterized by the up-regulation of osteoblastic markers, such as RUNX2 [6]. Our in vivo data showed that decreased ILK levels were involved in AoV calcification. Indeed, ILK silencing for 5 days increased RUNX2 expression, and ILK silencing for seven days also increased BPM2 expression, leading to calcification (Figure S1A). In vitro calcification assays require cell culture in pro-osteogenic conditions for at least 21 days; thus, we modified this assay to explore whether decreased ILK levels resulted in calcification. hVECs were cultured for eight days in pro-osteogenic medium and then SiRNA was transfected (scramble or ILK) for seven additional days, maintaining pro-osteogenic conditions. ILK silencing increased Alizarin Red staining and ILK overexpression three days after ILK silencing was able to reverse calcium nodule formation, suggesting that decreased ILK expression, particularly at the endothelial level, may contribute to the calcific process via EndMT (Figure S1B).

### 3.3. Nitric Oxide (NO) Prevents Osteogenic Differentiation Induced by ILK Silencing in hVECs

**Figure 3:**
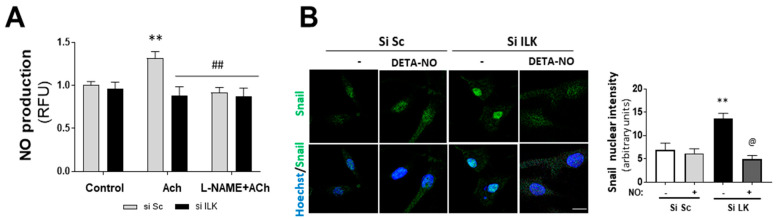

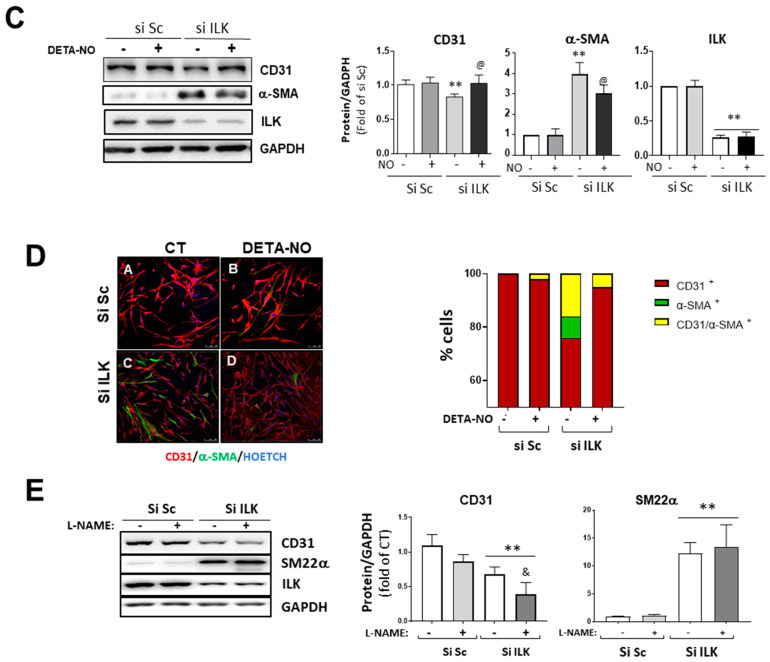
Nitric oxide prevents EndMT in hVECs. (**A**) Flow cytometry analysis of NO production in hVECs transfected with siSc or silk for 3 days. Cells were pre-treated with saline buffer (Control), Acetylcholine (ACh), L-NAME, or a combination thereof, according to the experimental procedure. n = 6. ** *p* < 0.001 vs. control; ^##^ *p* < 0.001 vs. Si Sc treated with Ach. (**B**) Confocal fluorescent microscopy images of SNAIL in transfected hVECs treated with the NO donor DETA-NONOate (DETA-NO) for 5 days (left). Nuclei were counterstained with Hoechst. Nuclear intensity quantification is represented on the right. Scale bar = 10 μm. n = 6. ** *p* < 0.001 vs. Si Sc; @ *p* < 0.001 vs. Si ILK without NO. (**C**) Western blot analysis (left) and quantification (right) of ILK, CD31, and alpha-smooth muscle actin (α-SMA) in transfected hVECs and treated with the NO donor DETA-NONOate (DETA-NO) for 5 days. n = 6. ** *p* < 0.001 vs. Si Sc; @ *p* < 0.001 vs. Si ILK without NO. (**D**) Double immunofluorescence of CD31 (red) and α-SMA (green) of hVECs in the same conditions as those in (A). Cell nuclei were counterstained with Hoechst. n = 10. Scale bar = 100 µm. (**E**) Western blot analysis (left) and quantification (right) of CD31 and alpha-smooth muscle actin (α-SMA) in transfected hVECs treated with the NOS inhibitor L-NAME for 5 days. n = 6. ** *p* < 0.001 vs. Si Sc; ^&^ *p* < 0.001 vs. Si ILK without L-NAME.

NO has been reported to inhibit spontaneous calcification in cultured porcine VICs (pAVICs) in vitro [17,18]. In addition, we previously reported that ILK indirectly interacts with eNOS, preventing eNOS from uncoupling in endothelial cells and ensuring adequate NO supply [10]. Therefore, we explored the role of NO production in EndMT induced by ILK silencing. First, we tested NO production of human VECs isolated from patients without calcification. As shown in Figure 3A, NO was increased in response to eNOS stimulation with Acetylcholine (Ach) and further prevented by the eNOS inhibitor L-NAME. However, ILK silencing for only three days decreased NO release in Ach-stimulated hVECs.

Silenced hVECs were treated with Apocynin to inhibit NADPH oxidase and were stimulated or not with ACh to test whether eNOS uncoupling was behind the decreased NO levels. ILK silencing triggered an increase in superoxide production as detected by confocal microscopy using DHE (Figure S2A). Accordingly, 3-nitrotyrosine (3-NT) expression, a stable biomarker of peroxynitrite production, increased in the aortic valves of the CAVD patients compared to the non-CAVD patients and in ILK-silenced hVECS (Figure S2B,C). Together, these results demonstrate that NO production in hVECs also depends on ILK expression and that an endothelial dysfunction is an early event after ILK expression decreases.

Then, we treated hVECs with an NO donor (DETA-NO) to mimic stable eNOS-NO release and studied EndMT induced by ILK silencing. DETA-NO treatment prevented Snail nuclear translocation, decrease in CD31, and increase in α-SMA at five days after ILK silencing, as detected by Western blotting and confocal microscopy (Figure 3B,C). After five days of siRNA transfection, 78 ± 2% cells conserved CD31, 5 ± 0.2% cells exhibited α-SMA and no CD31 expression, and the remaining 17 ± 3.2% expressed both markers, indicating an incomplete transition. In the presence of continuous NO supply, however, only a small fraction of cells (8 ± 0.2%) expressed both markers (Figure 3D). Next, we reasoned that if endothelial ILK effects on EndMT are produced via endothelial NO release, then decreasing endogenous NO production by eNOS inhibition with L-NAME would mimic ILK-silencing effects. L-NAME treatment induced a slight decrease in CD31 expression, further enhanced by ILK silencing. Similarly, L-NAME induced a modest increase in SM22α and potentiated ILK-silencing effects on SM22α expression (Figure 3E).

**Figure 4:**
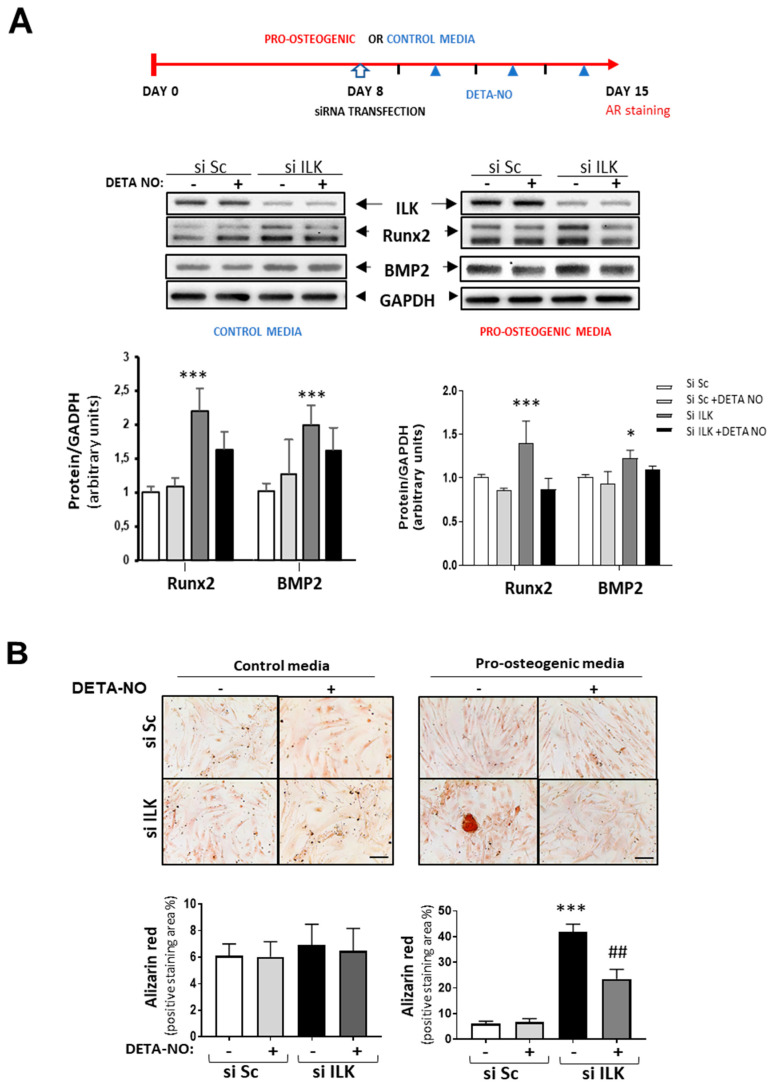
NO reverses the osteogenic phenotype induced by ILK silencing in hVECs. (**A**) Top: Scheme of the experimental design. Below: Western blot analysis (left) and quantification (right) of RUNX2 and BMP2. n = 8. * *p* < 0.05; *** *p* < 0.0001. (**B**) Alizarin Red staining. hVECs cultured with control medium or pro-osteogenic medium for 15 days were transfected with siSc or siILK for the last 7 days and treated with DETA-NO for 7 days. DETA-NO treatment was replenished every other day. Scale bar = 100 μm. Below: Quantification of alizarin red positive area. n = 6. Pro-calcific medium: *** *p* < 0.0001 vs. Si Sc; ^##^ *p* < 0.001 vs. Si ILK.

Interestingly, NO treatment prevented the increase in osteogenic markers already observed in ILK-silenced hVECs as compared with SiSc-transfected hVECs and the formation of calcified nodules after seven days in cells cultured under pro-osteogenic conditions but not in the control medium (Figure 4A,B).

Thus, collectively, our results show that NO prevented EndMT and osteogenic differentiation induced by ILK silencing.

### 3.4. NO Prevents ILK Silencing Effects on Valve Calcification through the TGF-β/Smad2/3 Signaling Pathway

We next analyzed the possible intracellular mechanisms by which ILK exerts its pro-osteogenic effects in hVECs. We examined the effects of ILK silencing on TGF-β and BMP signaling pathways which converge in RUNX2 to activate the transcription of osteogenic-related genes by canonical Smad-dependent pathways [19,20,21]. Western blot analysis with protein lysates from ILK-silenced hVECs revealed an increase in BMP2 levels in the absence of significant Smad1/5 phosphorylation by day 7 after ILK depletion, while RUNX2-increased expression was already detected as early as 5 days after silencing of ILK. At this time point, hVEC treatment with Noggin (500 ng/mL) (a potent BMP inhibitor that prevents BMP binding to cell-surface receptors [22]) did not cause any change in RUNX2 expression induced by ILK silencing, although Noggin treatment did reduce Smad1/5 phosphorylation, indicating an effective antagonism of BMP signalling. Treatment with the specific inhibitor of BMP2 receptor, LDN-193189 (500 nM), yielded the same result (Figure S3). These data suggest that the BMP2 signalling pathway may not be the critical factor involved in RUNX2-increased expression induced by decreased ILK levels.

**Figure 5:**
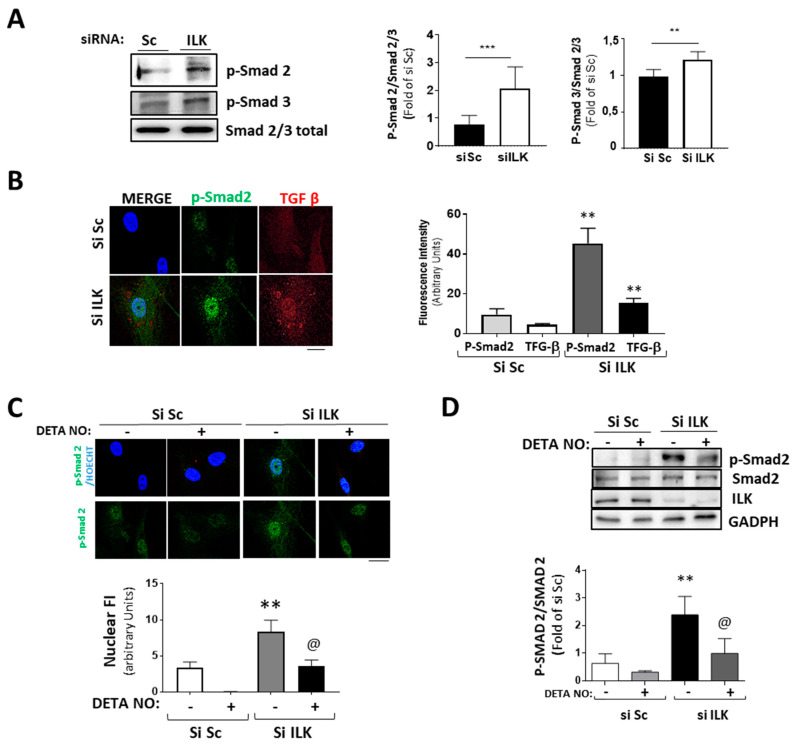
Silencing of ILK in hVECs induced EnMT through the TGF/SMAD2/3 axis in a nitric-oxide-dependent manner. (**A**,**B**) hVECs transfected with siSc or siILK for 5 days. (**A**) Western blot analysis and quantification of phospho-Smad2 and phospho-Smad3. *** *p* < 0.0001 vs. Si Sc; ** *p* < 0.001 vs. Si Sc (**B**) Immunofluorescence of phospho-Smad2 (green) and TGF-β (red) and quantification of fluorescence intensity (right). n = 6. ** *p* < 0.001 vs. Si Sc. (**C**,**D**) hVECs transfected with siSc or siILK and treated with DETA-NONOate for 5 days. Quantification of nuclear translocation (right). n = 6. ** *p* < 0.001 vs. Si Sc. (**C**) Confocal fluorescent microscopy images of phospho-Smad2. (**D**) Western blot analysis of phospho-Smad2 and quantification. n = 6. ** *p* < 0.001 vs. Si Sc; @ *p* < 0.001 vs. Si ILK.

The transforming growth factor β/Smad pathway can induce EndMT both in vivo and in vitro [5,6,23]. Five days of ILK silencing induced the phosphorylation and nuclear translocation of Smad2/3 and the expression of TGF-β, as detected by confocal microscopy (Figure 5A,B). Since NO antagonizes TGF-β responses in endothelial cells, as we previously demonstrated [24], and NO prevents the expression of osteogenic markers in ILK-silenced hVECs, we explored whether the NO effect was caused by interference with the TGF-β/Smad2/3 signaling pathway induced by ILK silencing in hVECs. Figure 5C,D show that NO reduced Smad2/3 nuclear translocation and phosphorylation in ILK silenced hVECs. Moreover, it also decreased the nuclear presence of Snail (Figure 3D), thus indicating a reduction in hVEC transformation potential in response to the loss of ILK.

**Figure 6:**
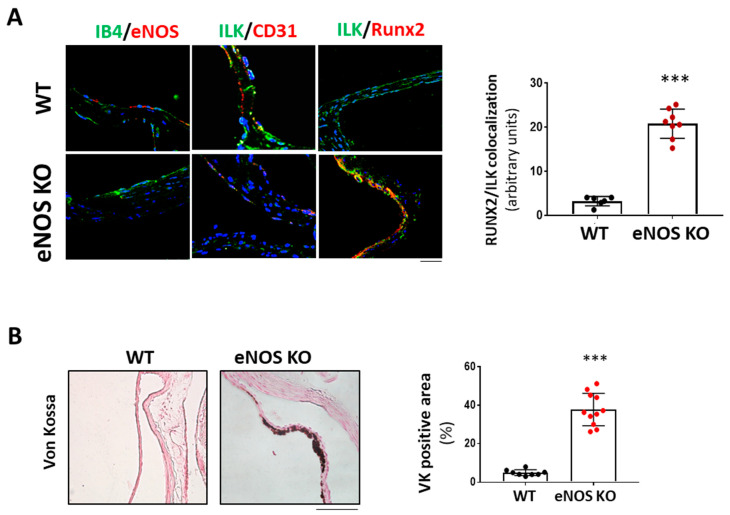
eNOS knock-out mice exhibited valve calcification, which correlates with low levels of ILK expression and increased RunX2 in valve endothelium. (**A**) Confocal microscopy image of endothelial marker IB4 in green and eNOS in red (left panel); ILK in green and endothelial marker CD31 in red (central panel); and ILK in green and Runx2 in red (right panel) in eNOS KO mice vs. WT. On the left, quantitation of ILK/RUNX2 colocalization in valve endothelium. N = 8. *** *p* < 0.0001 vs. WT. (**B**) Von Kossa staining in aortic valves of WT and eNOS KO mice. Scale bar = 50 μm. A quantification of VK positive area with respect to total area is shown below. n = 11. *** *p* < 0.0001 vs. WT.

We next hypothesized that if NO release mediated endothelial protective ILK effects, limiting endothelial NO release would mimic decreased ILK expression effects. We used eNOS KO mice as an animal model to demonstrate this point. As shown in Figure 6A, six-month-old eNOS KO mice presented increased RUNX2 expression and reduced endothelial ILK levels (33± 1.4%) relative to WT. Indeed, eNOS KO mice presented calcific deposition, as shown by Von Kossa staining, thus validating our hypothesis (Figure 6B). 

Together, those results strongly point to nitric oxide as a mediator of ILK effects by regulation of the TFG-β/Smad pathway and the prevention of the osteogenic transformation of hVECs.

## 4. Discussion

We established a novel role for ILK as a protective factor in CAVD. Using human calcified valves and human valvular endothelial cells, we demonstrated that decreased ILK levels in endothelial cells from the aortic side of human calcific aortic valves correlated with calcium burden. ILK expression prevents EndMT and inhibits the osteogenic transformation of endothelial valve cells. Moreover, we identified a mechanism by which decreased ILK contributes to CAVD. In the absence of ILK expression, valve endothelial cells do not produce eNOS-NO, thus triggering EndMT and enhancing RUNX2 expression, which could promote the osteogenic transformation of activated VECs.

One of the initial events in CAVD is inflammation that, together with mechanical stress, triggers endothelial dysfunction [3]. We observed that patients with CAVD expressed lower endothelial ILK levels than non-CAVD controls. The multivariant linear regression model showed that ILK expression is a protective factor in CAVD. BMP2 and RUNX2, two proteins strongly involved in valve calcification, were increased in CAVD compared to non-CAVD valves. Moreover, ILK levels showed a significant inverse correlation with RUNX2—a transcription factor closely involved in the osteogenic transformation of both VICs and VECs [3]. Oscillatory shear stress is very likely to be a contributing factor in CAVD. While the VECs from the ventricular surface area are exposed to linear high shear stress, VECs at the aortic surface are exposed to oscillatory low shear stress [25]. Accordingly, we observed a more pronounced decrease in ILK expression in VECs from the aortic side of CAVD patient valves, reinforcing the association of ILK with CAVD. Several limitations of this study are worth noting. First, we excluded patients with kidney failure, so our results cannot be extrapolated to this population. Second, we used non-CAVD as controls, and it would be desirable to perform a control group in healthy valves. Third, we only have data from surgically replaced valves with moderate to severe calcification, and mildly calcified valves should also be examined. Fourth, we excluded bicuspid valves from our study; it would be interesting to study whether the conclusions of this study also stand for this patient subgroup.

Strong evidence shows that VICs mediate the calcification process due to abnormal activation, osteogenic transformation, apoptosis, ECM remodeling, and calcium deposition. However, in recent years, several reports have suggested that valve endothelia could also contribute to CAVD. In the absence of disease, VECs release mediators that maintain VICs in a quiescent estate [26,27] and, by transforming into endothelial-derived VICs, VECs may replenish damaged VICs in the valve interstitium [28]. In a diseased state, VECs could experience dysregulated EndMT and osteogenic transformation, contributing to the progression of CAVD. The current study shows that human VECs responded to ILK silencing to elaborate an activated VIC phenotype. After five days of ILK silencing, cells expressed mesenchymal markers, and the EndMT characteristic disappearance of endothelial cell markers took place at seven days post-silencing. In addition, we observed endothelial cells expressing both markers inside the valves, suggesting the acquisition of endothelial migratory capabilities.

Moreover, mechanical strain can enhance EndMT in VECs via TGF-beta/Smad signaling cascades [29]. The osteogenic potential of clonal ovine VEC populations has also been demonstrated [30]. Our results show a negative correlation between decreased ILK levels and RUNX2 expression, suggesting a pro-osteogenic potential when ILK is downregulated. ILK silencing was sufficient to induce calcium nodule formation when cells were grown in pro-osteogenic medium, thus supporting the in vivo data. Interestingly, El-Hoss et al. have reported that the inactivation of ILK in osteoblasts increases mineralization [31].

The role of ILK as an essential mechanotransductor protein has been demonstrated in different tissues, such as fibroblasts during scar formation [32] and epithelial [33] and tumoral cells [34]. ILK is highly expressed in the cardiovascular system, transducing β1 integrin-dependent biomechanical stresses into contractile cells [35], and, as we previously demonstrated, ILK is part of a signaling platform that helps couple the mechanical signals received by the endothelial cells to the appropriate release of NO through its interaction with eNOS and Hsp90 [10]. Here, we have shown that, similar to vascular endothelial cells, ILK silencing in hVECs for three days impairs NO release and increases peroxynitrite formation. Moreover, valves from CAVD patients showed increased nitrotyrosine levels, indicative of enhanced nitrosative stress. We attempted to mimic eNOS-NO release by exposing ILK-silenced VECs to an NO donor. The NO donor prevented EndMT and decreased RUNX2 expression, while eNOS inhibition potentiated ILK-silencing effects in EndMT. In addition, eNOS KO mice exhibited increased RUNX2, valve calcification, and decreased ILK expression. This result expands our previous observation showing decreased endothelial ILK and valvular calcification in aged mice, suggesting that ILK may prevent valvular calcification through miRNA199-3p, which is related to NO synthesis [36]. Together, these results confirm the importance of ILK/NO in endothelial cell transdifferentiation.

NO signaling pathways can regulate VIC transformation and therefore influence CAVD development. NO can activate NOTCH1 signaling to inhibit calcification [18]. NO prevents CAVD by S-nitrosylation of USP9X, initiating a signaling pathway that culminates in the activation of NOTCH1, inhibiting RUNX2, thus preventing the calcification produced by VICs [17]. Recently, it has been described that patients with severe valvular stenosis have increased circulating metabolites related to NO metabolism and that the levels of these metabolites normalize when the valve is replaced [37]. In addition, endothelial BH4, a cofactor necessary for eNOS-induced NO production, can prevent osteoblastic differentiation of VICs [38]. We previously reported that ILK facilitates eNOS function by promoting BH4 bioavailability [10]. Thus, NO has been proposed as a protective factor in relation to VIC osteogenic transformation. Interestingly, our results are the first to show that the loss of ILK in the endothelium directly induces endothelial-to-mesenchymal transition in an NO-dependent manner. This process is directly linked to calcification, since NO prevents human VEC calcific nodule formation induced by ILK silencing. Furthermore, eNOS KO mice showed reduced ILK and increased RUNX2 levels in their aortic valves, thus confirming the role of ILK in CAVD.

Interestingly, we found a correlation between decreased ILK levels and BMP2 expression. However, our experimental data prompted us to consider that the BMP2 pathway may not be the main effector of osteogenic transformation induced by ILK silencing. Indeed, some reports suggested that BMP signaling may not be sufficient for VIC calcification [39]. A correct balance between bone morphogenetic protein (BMP) and transforming growth factor-beta (TGFβ) signaling is equally relevant for valve homeostasis, and a switch of this balance toward TGFβ signaling is a hallmark of endothelial cell (EC) dysfunction [40].

Endothelial responses to TGFβ include extracellular matrix (ECM) production and endothelial-to-mesenchymal transition (EndMT) through Smad-dependent and Smad-independent signaling pathways [41]. Here, we have shown that ILK silencing results in Smad2/3 activation and TGF-β expression. Importantly, mechanically challenged VICs became hyper-responsive to TGF-β, elaborating a myofibroblast phenotype and osteogenic potential [42]; thus, endothelial ILK decreased expression could enhance the osteogenic potential of VICs by increasing TGF-β/Smad signaling. The role of ILK in TGF-β signaling in the context of EndMT has not been explored before, but TGF-β signaling can activate epithelial-to-mesenchymal transition (EMT), a process following the basic principles of EndMT, and ILK participates in TGF-EMT [43]. There may be several explanations for this apparent contradiction. First, EndMT mechanisms may be cell-context-specific. Notably, most of the research on the cellular cascades involving EMT triggered by ILK has been performed on transformed cells or tumor models [31,44,45]. Second, the experimental design followed in those studies was different, relying on a triggering stimulus, such as periostin, TGF-beta, or hypoxia, to study the signaling pathways involved, while our study shows the effect of a decrease in ILK levels [43,46,47,48]. Third, ILK expression in the cardiovascular system is mainly cardioprotective, as explained above; thus, a slight reduction in its expression levels results in dramatic pathological changes, EndMT being one process involved in cardiovascular pathologies [49]. Thus, although additional studies will be necessary to dissect the molecular pathways involved in the transdifferentiation program initiated by ILK depletion, our study is the first to show the involvement of ILK in EndMT in the setting of CAVD development. However, the involvement of other pathways, such as the Wnt/β-catenin pathway and Notch signaling, cannot be discounted [50]. Nevertheless, the TGF-β/Smad2 pathway has been linked to RUNX2 expression in calcific valve disease [51] and mesenchymal cells [52]. In addition, RUNX2 binds to its promoter via OSE2, a cis-acting element initially identified as a RUNX2-binding site on the osteocalcin promoter, and regulates its own expression [53]. Thus, ILK silencing could initiate a feedforward cycle of RUNX2 expression, influencing VEC osteoblastic fate.

Our results show that NO can inhibit EndMT induced by decreased ILK endothelial levels. We previously described that NO could regulate TFG-β responses in vascular endothelial cells by decreasing the stability of Smad2 [24]. Here, we have shown that NO can inhibit Smad2/3 phosphorylation and decrease the nuclear translocation of Snail1. Moreover, NO can inhibit RUNX2-increased expression induced by ILK. TGF-β/Smad signaling regulation is complex and can be modulated at several levels. It is possible to speculate that NO may regulate Smad2 stability or even induce the expression of the inhibitories Smad6 and 7. In a hypercholesterolemia-driven mouse model of CAVD, cholesterol-lowering strategies reduced calcification but not fibrosis or Smad2 signaling, and this effect was attributed to the selective down-regulation of Smad6 [54].

## 5. Conclusions

This study demonstrated that ILK endothelial expression is strongly associated with human CAVD development. ILK exerts a protective role in CAVD by preventing valvular endothelial dysfunction and osteogenic transformation initiated by endothelial cell phenotypic transition. Thus, ILK might be a new therapeutic target in preventing aortic valve calcification during CAVD.

## Data Availability

Data are contained within the article and Supplementary Materials.

## Supplementary Materials

The following supporting information can be downloaded at: https://www.mdpi.com/article/10.3390/antiox11091736/s1: Table S1: Reagents and Antibodies, Table S2: Patient demographic and clinical characteristics, Figure S1: Silencing of ILK in human valve endothelial cells increased osteogenic markers, Figure S2: ILK silencing in hVECs impaired nitric oxide production by eNOS, Figure S3: Inhibition of the BMP2 pathway in ILK-silenced hVECs did not restore RUNX2 levels.
