# Supplementary material for: Integrin-Linked Kinase Expression in Human Valve Endothelial Cells Plays a Protective Role in Calcific Aortic Valve Disease
Source: Antioxidants (Basel). 2022 Aug 31;11(9):1736. doi: 10.3390/antiox11091736 (PMC9495882; doi:10.3390/antiox11091736)
Supplement: Supplementary file 1 [file antioxidants-11-01736-s001.zip › antioxidants-1869618-figure.pdf]

FIGURE S1

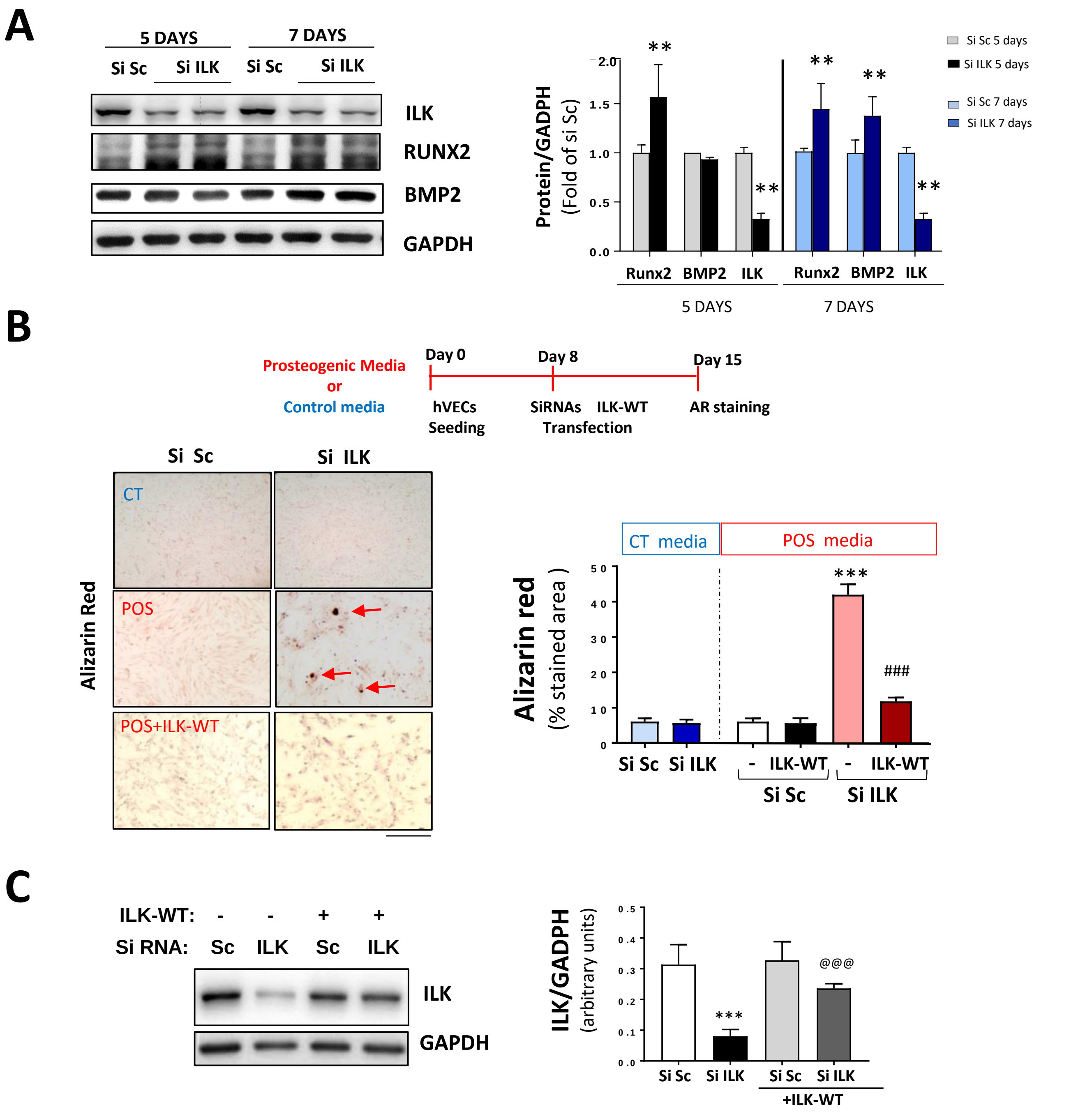

Figure S1. Silencing ILK in human valve endothelial cells increases osteogenic markers. A) Western blot analysis and quantification of RUNX2 and BMP2 in hVECs transfected with siRNA Scramble (Si Sc) or siRNA ILK (Si ILK) for 5 and 7 days. n=7; **p<0.001 vs Si Sc. hVECs were cultured in endothelial cell media **B)** Upper panel: experimental design of the experiment. Left lower panel: Alizarin Red staining of hVECs cultured with control (CT) or pro-osteogenic (POS) media for 15 days and transfected with siSc or siILK the last seven days. hVECS were transfected with V5-tagged ILK-WT three days after ILK silencing. Scale bar= 100 µm.

Right lower panel: Quantification of alizarin red staining (n=8). ***p<0.0001 vs. Si Sc in POS media; ###p<0.001 vs. Si ILK Left lower panel. **C)** Representative western blot of ILK expression in cells treated as in B) (n=6). ***p<0.0001 vs. Si S; @@@p<0.001 vs. Si ILK.

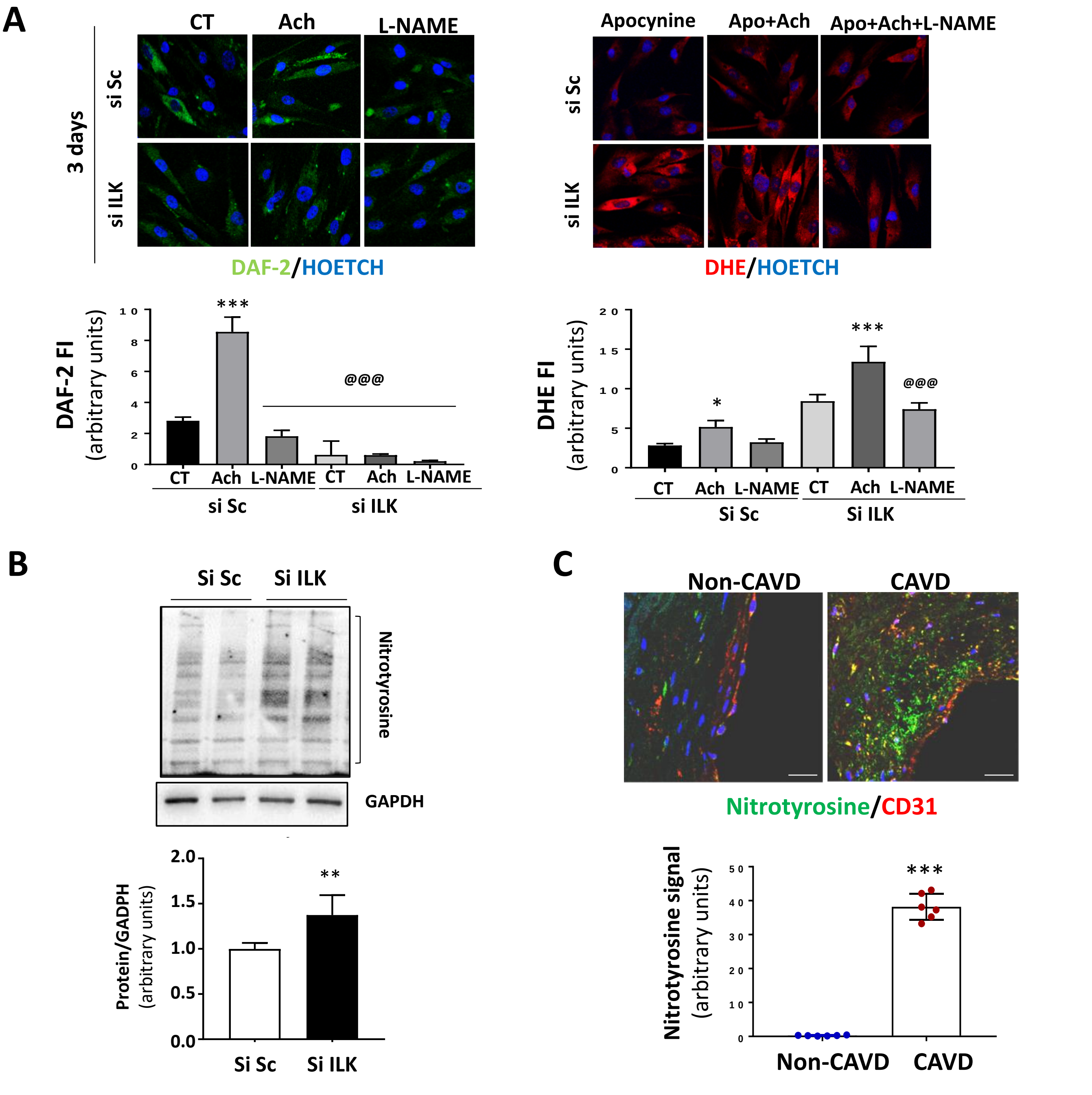

Figure S2. ILK silencing in hVEC impairs nitric oxide production by eNOS. A) Confocal microscopy images of DAF-2 probe (left panel) or DHE probe (right panel) in hVECs transfected with siSc or silk for 3 days. Cells were pre-treated with saline buffer (CT), Acetylcholine (Ach), L-NAME, Apocynin (Apo) or in combination according to the experimental procedure. Scale bar= 10 µm. Below: quantification of fluorescence intensity n=6; ***p<0.0001 vs. Si Sc, @@@p<0.0001 vs Si ILK. **B)** Western blot analysis (up) and quantification (down) of nitrotyrosine formation in siSc or siILK hVEC. n=4; *p<0.05 **C)** Immunofluorescence of nitrotyrosine (green) and CD31 (red) in non-CAVD or CAVD human aortic valve leaflets. Nuclei were counterstained with Hoechst. n=6, scale bar= 25 µm. ***p<0.001.Below: quantification of nitrotytorisne. N=6 ***p<0.0001.

FIGURE S3

A

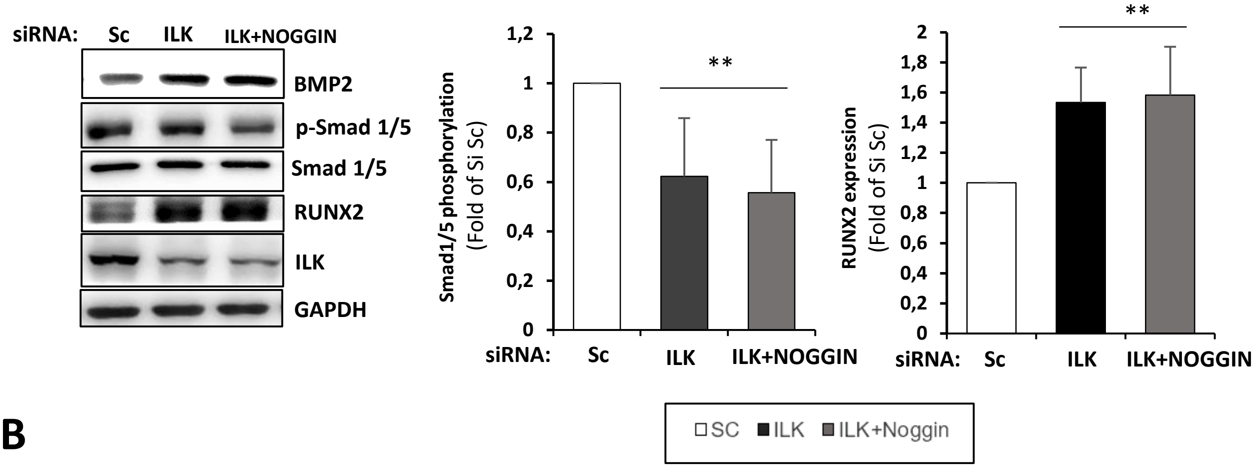

B

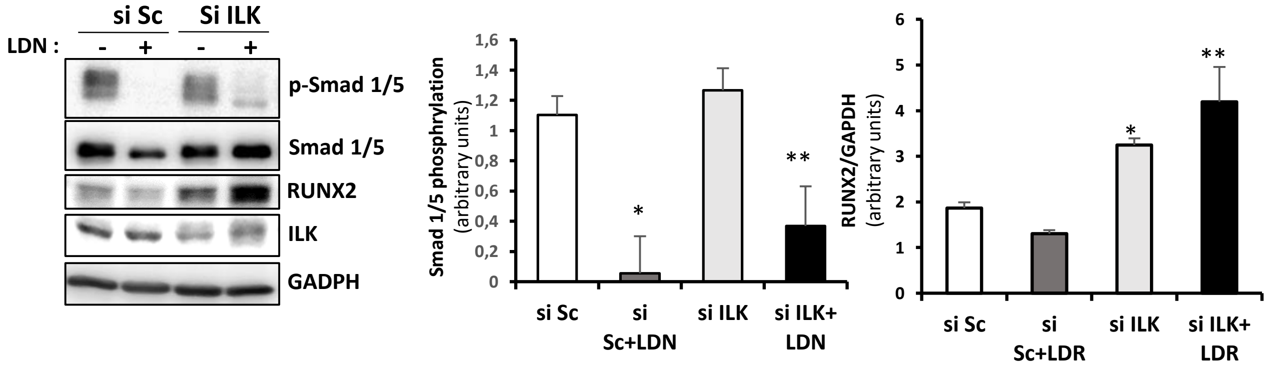

Figure S3. Inhibition of BMP2 pathway in ILK-silenced hVEC does not restore Runx2 levels. A-B) Western blot (left) and quantification (right) of BMP2/Smad1-5/Runx2 axis in siSc or siILK hVEC treated with **A)** Noggin, a BMP2 agonist (n=6; **p<0.001) or **B)** LDN-193189 (LDN), a selective BMP signaling inhibitor (n=3; *p<0.01 vs siSC; **p<0.001 vs si ILK).
