# Supplementary material for: Integrin-Linked Kinase Expression in Human Valve Endothelial Cells Plays a Protective Role in Calcific Aortic Valve Disease
Source: Antioxidants (Basel). 2022 Aug 31;11(9):1736. doi: 10.3390/antiox11091736 (PMC9495882; doi:10.3390/antiox11091736)
Supplement: Supplementary file 1 [file antioxidants-11-01736-s001.zip › SUPPLEMENTARY TABLE S1.pdf]

Table S1. Reagents and Antibodies.

Carazzi hematoxylin and DPX were from Casa Alvarez (Madrid, Spain). Mouse and Rabbit Specific HRP/DAB (ABC) Detection IHC kit and Von Kossa Stain Kit were from Abcam. General cell culture supplies were purchased from Lonza (Basel, Switzerland); fetal bovin serum was from Gibco (Waltham, Ma, USA); matrigel from Corning (Corning, NY, USA). Cell culture-grade gelatin, hematoxylin-eosin, Alizarin Red, beta-glycerolphosphate, ascorbic acid, AcH and Apocynin from Sigma Aldrich (San Luis, MO, USA). SuperSignal detection system Pierce™ ECL Western Blotting Substrate and Noggin from Thermo Fisher Scientific (Waltham, MA,

USA). Dihydroethidium (DHE), DAF-2, DETA-NONOate, and Dexamethasone from Invitrogen Corporation (Carlsbad, CA, USA). Complet mini, phosphostop, Dispasa II were from Roche (Switzerland). LDN193189 and Fluorsave Reagent from Merck (Darmstadt, Germany). A detail listing of the antibodies used through the study is provided bellow.

Antibody	Supplier	Reference	Dilution
**Anti-ILK**	RD Systems	MAB374	WB: 1-1000
	Bioss	BS-0317R	IF/IHC: 1-50
**Anti-Ve-cadherin**	Santa cruz	sc-9989	WB: 1-250
**Anti-CD31**	Abcam	ab32457	IF: 1-50<br>WB:1-500
**Anti- a-smooth muscle actin**	Thermo Scientific	MA1-06110	IF:1-200 WB:1-1000
**Anti-GAPDH**	Millipore	MAB374	WB: 1-1000
**Anti- Transgelin**	Santa Cruz	sc-53932	WB: 1-1000
**Anti- SNAI**	Santa Cruz	sc-271977	WB: 1-1000<br>IF: 1:50
**Anti- phospho Smad 2**	Cell signaling	18338	WB: 1-500<br>IF: 1-50
**Anti- phospho Smad 3**	Santa Cruz	sc-517575	WB: 1-500
**Anti- von Willebran Factor**	Santa Cruz	sc-365712	WB: 1-500
**Anti-TGFβ**	Santa Cruz	sc-130348	IF:1-50
**Anti-Smad2/3**	BD Biosciences	610842	WB: 1-500
**Anti-BMP2**	Abcam	ab82511	WB: 1-1000
**Anti- RUNX2**	Santa Cruz	Sc-390351	WB: 1-500<br>IF:1-50
**Anti- phospho Smad 5**	Cell signaling	9516	WB: 1-500
**Anti-Smad 5**	Cell signaling	12534	WB: 1-500
**anti-GSl I-isoleoctin B4 (IB4)**	Vector Laboratories	FL-1201	IF:1-100
**eNOS**	BD Biosciences	610296	IF:1-50
**Anti-Nitrotyrosine**	Millipore	06-284	WB:1-1000<br>IF: 1-50
**HRP-conjugated anti-Mouse secondary antibody**	Invitrogen	A16072	WB: 1-20000
**HRP-conjugated anti-Rabbit secondary antibody**	Invitrogen	31466	WB: 1-50000
**Alexa fluor 488 conjugated Goat anti-Rabbit secondary antibody**	Abcam	ab150081	IF,:1-500
**Alexa fluor 488 conjugated Goat anti-mouse secondary antibody**	Abcam	ab150113	IF,:1-500

**Alexa fluor 647 conjugated Goat anti-mouse secondary antibody**	Abcam	ab150115	IF,:1-500
**Alexa fluor 647 conjugated Goat anti-Rabbit secondary antibody**	Abcam	ab150083	IF,:1-500
