# Supplementary material for: Integrin-Linked Kinase Expression in Human Valve Endothelial Cells Plays a Protective Role in Calcific Aortic Valve Disease
Source: Antioxidants (Basel). 2022 Aug 31;11(9):1736. doi: 10.3390/antiox11091736 (PMC9495882; doi:10.3390/antiox11091736)
Supplement: Supplementary file 1 [file antioxidants-11-01736-s001.zip › SUPPLEMENTARY TABLE S2.pdf]

Table S2: List of most relevant clinical characteristics and preoperative comorbidities

PREOPERATIVE CLINICAL AND CARDIOLOGIC CHARACTERISTICS AND COMORBIDITIES		
**Variables**	**CAVD**	**NON-CAVD**
Age (years), mean (SD)	67,73 (9,20)	66,37 (10,68)
Male sex, n (%)	53 (70,67%)	19 (67,86%)
Diabetes mellitus, n (%)	23 (30,67%)	4 (14,29%)
Dyslipidemia, n (%)	44 (58,67%)	10 (35,71%)
Smoking, n (%)	23 (30,67%)	12 (42,86%)
Arterial hypertension, n (%)	49 (65,33%)	21 (75%)
Peripheral vascular disease, n (%)	11 (14,67%)	8 (28,57%)
Chronic obstructive pulmonary disease (COPD), n (%)	14 (18,76%)	3 (10,71%)
Pre-operative atrial fibrillation, n (%)	17 (22,67%)	7 (25%)
Coronary artery disease, n (%)	13 (17,33%)	3 (10,71%)
Concomitant mitral valve disease, n (%)	14 (18,66%)	1 (3,57%)
Concomitant tricuspid valve disease, n (%)	5 (6,66%	1 (3,57%)
Concomitant aortic disease, n (%)	8 (10,66%)	10 (35,71%)
LV systolic dysfunction	29 (38,66%)	21 (75%)
Preoperative congestive heart failure, n (%)	11 (14,66%)	4 (14,28%)
NYHA III-IV, n (%)	37 (49,33)	14 (50%)

SD: standard deviation; NYHA: New York Heart Association; LV: left ventricle; LV: left ventricle.
